# Lung flooding enables efficient lung sonography and tumour imaging in human *ex vivo* and porcine *in vivo* lung cancer model

**DOI:** 10.1186/2047-783X-18-23

**Published:** 2013-07-10

**Authors:** Thomas Günther Lesser, Harald Schubert, Sabine Bischoff, Frank Wolfram

**Affiliations:** 1 Department of Thoracic and Vascular Surgery, SRH Wald-Klinikum Gera, Teaching Hospital of Friedrich-Schiller University of Jena, Strasse des Friedens 122, Gera D-07548, Germany; 2 Institute of Animal Experimentation, Friedrich-Schiller University of Jena, Bachstrasse 18, Jena D-07743, Germany

**Keywords:** Carcinoma, Endoscopic lung surgery, Lung cancer, Lung tumour, Lung flooding, Lung sonography, Simulated lung tumour, Tumour detection, Tumour imaging

## Abstract

**Background:**

Sonography has become the imaging technique of choice for guiding intraoperative interventions in abdominal surgery. Due to artefacts from residual air content, however, videothoracoscopic and open intraoperative ultrasound-guided thermoablation of lung malignancies are impossible. Lung flooding is a new method that allows complete ultrasound imaging of lungs and their tumours.

**Methods:**

Fourteen resected tumourous human lung lobes were examined transpleurally with B-mode ultrasound before (in atelectasis) and after lung flooding with isotonic saline solution. In two swine, the left lung was filled with 15 ml/kg isotonic saline solution through the left side of a double-lumen tube. Lung tumours were simulated by transthoracic ultrasound-guided injection of 5 ml of purified bovine serum albumin in glutaraldehyde, centrally into the left lower lung lobe. The rate of tumour detection, the severity of disability caused by residual gas, and sonomorphology of the lungs and tumours were assessed.

**Results:**

The *ex vivo* tumour detection rate was 100% in flooded human lung lobes and 43% (6/14) in atelectatic lungs. In all cases of atelectasis, sonographic tumour imaging was impaired by residual gas. Tumours and atelectatic tissue were isoechoic. In 28% of flooded lungs, a little residual gas was observed that did not impair sonographic tumour imaging. In contrast to tumours, flooded lung tissue was hyperechoic, homogeneous, and of fine-grained structure. Because of the bronchial wall three-laminar structure, sonographic differentiation of vessels and bronchi was possible. In all cases, malignant tumours in the flooded lung appeared well-demarcated from the lung parenchyma. Adenocarcinoma, squamous, and large cell carcinomas were hypoechoic. Bronchioloalveolar cell carcinoma was slightly hyperechoic. Transpleural sonography identifies endobronchial tumour growth and bronchial wall destruction. With transthoracic sonography, the flooded animal lung can be completely examined *in vivo*. There is no residual gas, which interferes with ultrasound. Pulmonary vessels and bronchi are clearly differentiated. Simulated lung lesions can easily be detected inside the lung lobe.

**Conclusions:**

Lung flooding enables complete lung sonography and tumour detection. We have developed a novel method that efficiently uses ultrasound for guiding intraoperative interventions in open and endoscopic lung surgery.

## Background

In cases of inoperable primary or metastatic liver tumours, radiofrequency or microwave ablation are accepted therapeutic options [1-3]. As a less invasive therapy, ablation can be performed under percutaneous, laparoscopic, or open intraoperative ultrasound guidance [4-7]. Lung tumours in the parenchyma are undetectable by ultrasound due to artefacts caused by residual air content. Because of this, transthoracic, videothoracoscopic, or open intraoperative ultrasound-guided interstitial thermotherapy for treating non-resectable primary and secondary lung tumours is impossible. Lung flooding could be a new method to image the lung and tumours completely with ultrasound. This study aimed to investigate lung sonography and tumour imaging under flooding conditions, *ex vivo* on resected human lung lobes and *in vivo* in a porcine model.

## Methods

### *Ex vivo* examinations of resected human lung lobes

#### Human lung samples

Between April 2011 and March 2012, 14 patients with lung tumours were enrolled. This study was approved by the ethics committee of the Medical Association of Thuringia. All patients received a lung lobectomy. In 13 cases, presurgical histological diagnosis of a malignant tumour was confirmed using a percutaneous or transbronchial needle biopsy (10 bronchial carcinomas, one lung metastasis of a colon carcinoma, one peribrochial lymph node metastasis of a thyroid carcinoma, and one peribrochial lymph node metastasis of a renal cell carcinoma). Due to a central hamatochondroma, one case also required middle lobe resection. The average age of the patients was 67.5 years (range 54 to 85 years), from whom the right lower lobe (n = 6), right upper lobe (n = 4), left lower lobe (n = 2), or the middle lobe (n = 2) were resected. The greatest tumour diameter, determined by computed tomography scan, was 3.2 cm (range 1.6 to 8.0 cm).

#### Intraoperative procedures

Lungs were ventilated with 100% oxygen after intubation with a double-lumen endotracheal tube. After anterolateral thoracotomy in the lateral decubitus position, the segmental pulmonary arteries and lobar vein were dissected, parenchymal fusions between the lobes were cut with staplers, and the lobar vein and secondary segmental pulmonary arteries were divided. A mediastinal lymphadenectomy was carried out before dividing the lobar bronchus to achieve complete atelectasis of the lung lobe.

#### Lung flooding

**Figure 1:**
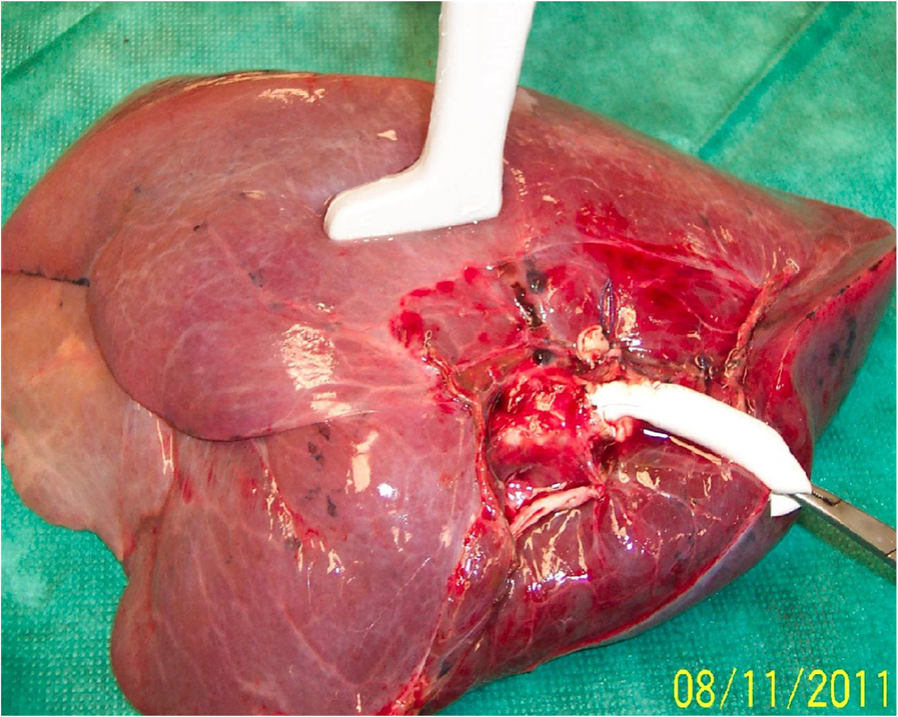
**Sonographic examination of a completely saline-flooded right inferior pulmonary lobe.** A conduit anastomosed end-to-end to the inferior lobar bronchus allowed filling with saline completely.

The resected lobe was prepared for flooding immediately *ex vivo*, and mucus in the segmental and subsegmental bronchi was removed. An expanded polytetrafluoroethylene graft was anastomosed end-to-end to the lobar bronchus and used as a conduit for fluid instillation. After venting the conduit, an infusion system was connected. Filling was performed passively using the gravity of the liquid flowing from an infusion bottle suspended 50 cm above heart level. Filling continued until functional residual capacity of the lobe was achieved (Figure 1).

#### Sonographic examination

Before and after liquid filling, the lung lobe was examined transpleurally by ultrasound (MikroMaxx™ Portable Ultrasound System; SonoSite, Inc., Bothell, WA, USA) in a liquid bath of 30°C isotonic NaCl solution with a linear probe (L 38e, 10 to 5 MHz; SonoSite) and an intraoperative probe (SLA, 13 to 6 MHz; SonoSite) in fundamental B-mode. The surgeon was not the ultrasound examiner. The investigator did not know the preoperative computed tomographic findings. We assessed tumour detection rate, the imaging disability caused by residual gas, and the sonomorphology of the lung and the tumour, and their spatial relationships to the bronchi and pulmonary vessels.

Histopathological examinations of the resection margins were performed immediately after the ultrasound. For the definitive histopathological examination, the specimen was fixed in formaldehyde after a cut through the lobe.

### *In vivo* ultrasound detection of simulated tumours in a porcine model

#### Animals

Animal experiments were carried out on two female pigs (Deutsches Landschwein breed; weight range: 33 to 38 kg, average: 35.5 kg), with permission from the Veterinary Department of the Thuringian State Authority for Food Protection and Fair Trading, and in compliance with the National Animal Protection Act.

#### Anaesthesia and artifical respiration

Anaesthesia was induced by intramuscular injection of 10 mg/kg^-1^ ketamine. Additionally, 6.25 mg droperidol and 10 mg diazepam were administered after cannulation of an ear vein, and the animals were orotracheally intubated during spontaneous breathing (Magill tube, inner diameter = 8.5 mm, Mallinckrodt™, Covidien, Neustadt, Germany). After relaxation with pancuronium bromide (0.2 mg/kg^-1^) and deepening of the anaesthesia by fentanyl (10 μg/kg^-1^), artificial respiration was started with 1.0 to 1.5 minimum alveolar concentration (MAC) of isoflurane in an oxygen/nitrous oxide mix (fraction of inspired oxygen, FIO2 = 0.3). After tracheotomy, a left-sided Robertshaw double-lumen tube with an extra-long bronchial lane (size 39 Ch; special product by Mallinckrodt Medical, Dublin, Ireland) was inserted. The correct position of the tube was checked by fibre bronchoscopy (BF 3C30 Fiber Bronchoscope; Olympus, Tokyo, Japan). Anaesthesia was changed to total intravenous anaesthesia with propofol (10 mg/kg/h), fentanyl (0.05 to 0.08 μg/kg^–1^/min^–1^), and pancuronium bromide (2.5 μg/kg^–1^/min^–1^), and the FIO2 was raised to 1.0.

Mechanical ventilation was performed with an ICU respirator (Servo 900, Siemens AG, Munich, Germany), using a volume-controlled setting (tidal volume 10 ml/kg^-1^; respiratory rate 16 to 20 min^-1^; positive end-expiratory pressure = 6 cm H_2_O). The end-expiratory carbon dioxide partial pressure (pCO_2_) was maintained between 35 and 45 mmHg. We infused 4 to 6 ml/kg/h Ringer’s lactate and 2 to 4 ml/kg/h hydroxyethyl starch (HES 10%) as base infusions. Body temperature was maintained between 36 and 38°C by warming the infusion solution and covering the animals with an isolation sheet.

The electrocardiogram, arterial blood pressure, capillary oxygen saturation, and expiratory CO_2_ concentration were measured and recorded continuously (Datex AS/3 Compact Multiparameter Patient Monitor; Datex-Ohmeda Corp., Helsinki, Finland). Arterial blood gas samples were analysed every 30 minutes (ABL System 625; Radiometer Medical, Copenhagen, Denmark).

#### Lung flooding

Thirty minutes after ventilation with FIO_2_ = 1.0, the left endobronchial leg was disconnected from the respirator. The infusion system was immediately connected to the left tube leg and the lung was slowly filled with 15 ml/kg (accordingly the functional residual capacity of a lung wing) of an isotonic saline solution, preheated to body temperature. A single filling was performed passively using the gravity of the liquid flowing from an infusion bottle suspended 50 cm above heart level. The liquid was left in the lung for 30 minutes. During unilateral lung ventilation, the respirator settings remained unchanged.

Thirty minutes after flooding and completion of the sonographic examinations, the liquid was drained passively through the opened left tube leg after placing the animals in the Trendelenburg position (posterior of animal elevated 30°), followed by simultaneous ventilation of both lungs. After 30 minutes of two-lung ventilation, the animal was killed by injection of a lethal dose of sodium pentobarbital and potassium chloride.

#### Tumour simulation

After lung flooding, a 17-G needle was placed centrally in the left lower lung lobe, using percutaneous transpleural ultrasound guidance. Five millilitres of a fluid composed of purified bovine serum albumin and glutaraldehyde (Bioglue™; CryoLife Europa, Guildford, UK) was injected to simulate a lung tumour.

#### Lung sonography

**Figure 2:**
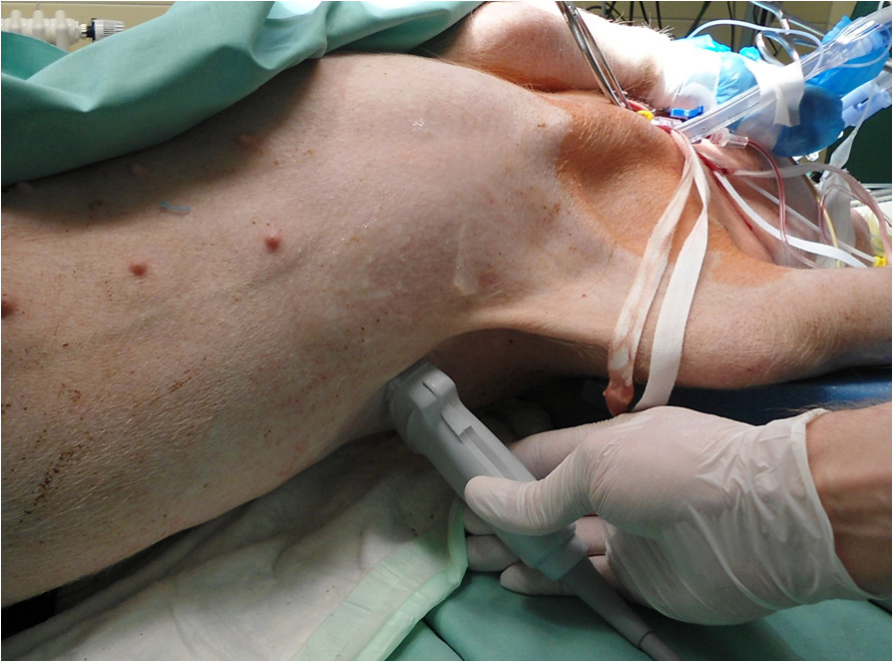
**Transthoracic sonography examination of a pig left lung after *in vivo *flooding with saline solution.** Unilateral lung flooding using a double-lumen tube and percutaneous transthoracic application of an ultrasound probe resulted in excellent visualisation of lung structure.

After liquid filling and tumour simulation, the left lung was examined transthoracically and transpleurally with ultrasound (MikroMaxx System; SonoSite, Inc.) with a linear (L 38e, 10 to 5 MHz) and curved probe (C11e, 8 to 5 MHz), in fundamental B-mode (Figure 2). We assessed the detection of simulated lesions and the sonomorphology of the lung.

## Results

### *Ex vivo* examination of resected human lung lobes

#### Sonomorphology of the lungs and tumours

**Figure 3:**
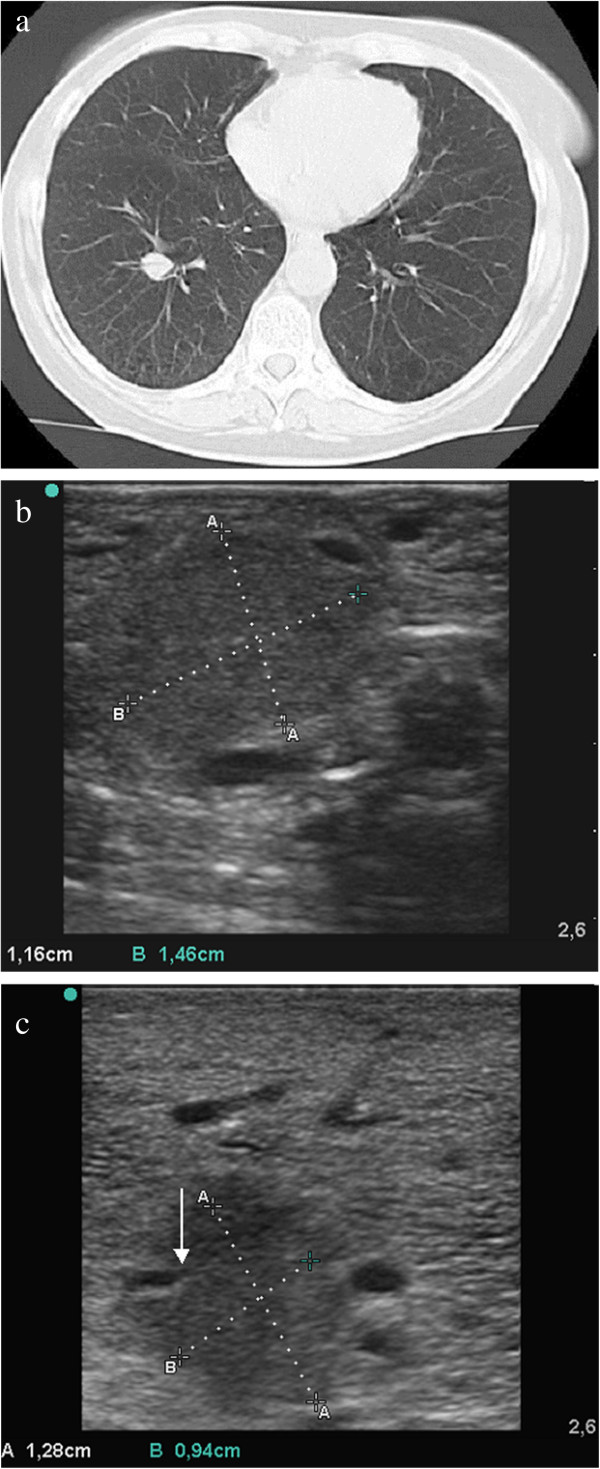
**Adenocarcinoma in the central right inferior lobe of an excised human lung. (a)** Computed tomography scan shows a central, peribrochial tumour. **(b)** Sonography under atelectasis: the tumour appears homogenous and is poorly demarcated (isoechogenic) from lung tissue. **(c)** Sonography after flooding: homogenous tumour tissue is hypoechoic, irregularly configured with fringe-like processes, and is well-demarcated from the normal lung parenchyma. Bronchial destruction is evident (arrow).

The tumour detection rate and sonomorphology are detailed in Table 1. Sonographic examination of the atelectatic lung was greatly limited by residual gas, whereby tumours were only detectable in 43% (6/14) of the cases. The detected tumours could be clearly demarcated from the surrounding lung tissue in only 15% of the cases. Tumours and atelectatic tissue presented as isoechoic, making distinction difficult (Figure 3).

**Table 1:** Sonographic examination of lung tumours before and after lung flooding

**Patient gender, age (years)**	**Tumour location**	**Size, mm (by CT)**	**Histology**	**Detected with atelectasis**	**Detected after flooding**	**Sonomorphology**	**Residual gas after flooding**
m (54)	RLL, peripheral	80	Adenocarcinoma	Yes	Yes	Hypoechoic, homogeneous	Small
m (65)	RUL, central	20	LNM thyroid cancer	No	Yes	Hypoechoic, homogeneous	No
m (69)	LLL, central	30	Squamous cell carcinoma	Yes	Yes	Hypoechoic, inhomogeneous	No
m (66)	RUL, central	35	Adenocarcinoma	No	Yes	Hypoechoic, homogeneous	No
f (54)	RLL, central	40	Adenocarcinoma	Yes	Yes	Hypoechoic, inhomogeneous	No
f (85)	ML, peripheral	30	Adenocarcinoma	Yes	Yes	Hypoechoic, homogeneous	Small
f (77)	RLL, central	20	Squamous cell carcinoma	No	Yes	Hypoechoic, inhomogeneous	Small
m (70)	ML, central	25	Chondroma	No	Yes	Complex, coarse-grained	No
m (60)	RLL, central	26	Squamous cell carcinoma	No	Yes	Hypoechoic, inhomogeneous	No
f (68)	RLL, central	16	Bronchioloalveolar cell carcinoma	No	Yes	Hyperechoic, homogeneous	No
f (64)	RUL, central	24	LNM renal cell carcinoma	No	Yes	Hypoechoic, homogeneous	No
m (75)	LLL, peripheral	21	Colon metastasis	Yes	Yes	Hypoechoic, homogeneous	No
m (67)	RUL, peripheral	25	Adenocarcinoma	No	Yes	Hypoechoic, homogeneous	No
m (71)	RLL, central	63	Large cell carcinoma	Yes	Yes	Hypoechoic, inhomogeneous	Small

Fourteen resected human lung lobes were assessed for tumour detection rate, sonomorphology, and limitations due to residual gas. *CT* Computed tomography, *LLL* Left lower lobe, *LNM* Lymph node metastasis, *ML* Middle lobe, *RLL* Right lower lobe, *RUL* Right upper lobe.

**Figure 4:**
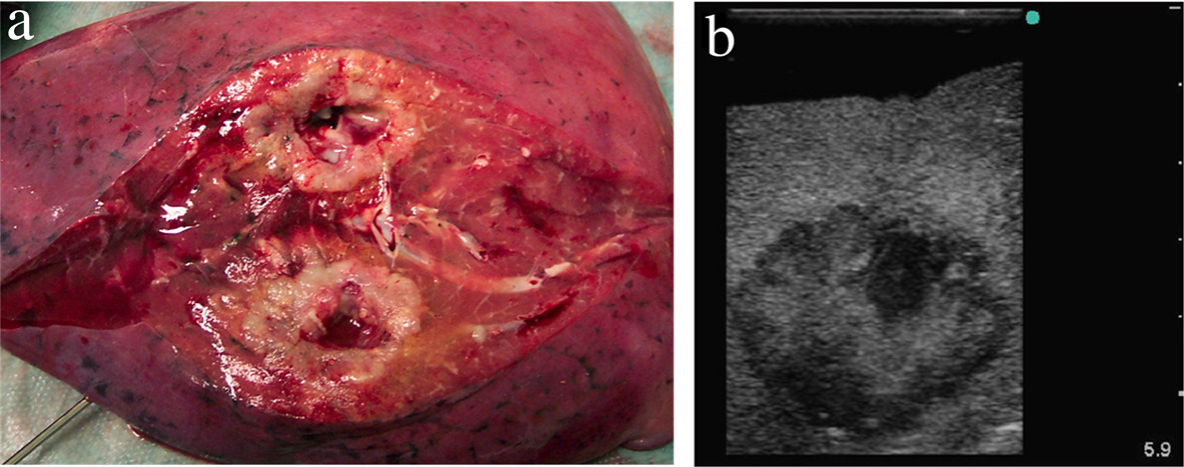
**Sonomorphology of a squamous cell carcinoma with central necrosis in a flooded human lung lobe. (a)** Macroscopic specimen (cut through the tumour after sonographic examination). **(b)** Sonography of the tumour shows its inhomogeneous texture with hypoechoic necrotic areas and a hypoechoic ‘halo’.

**Figure 5:**
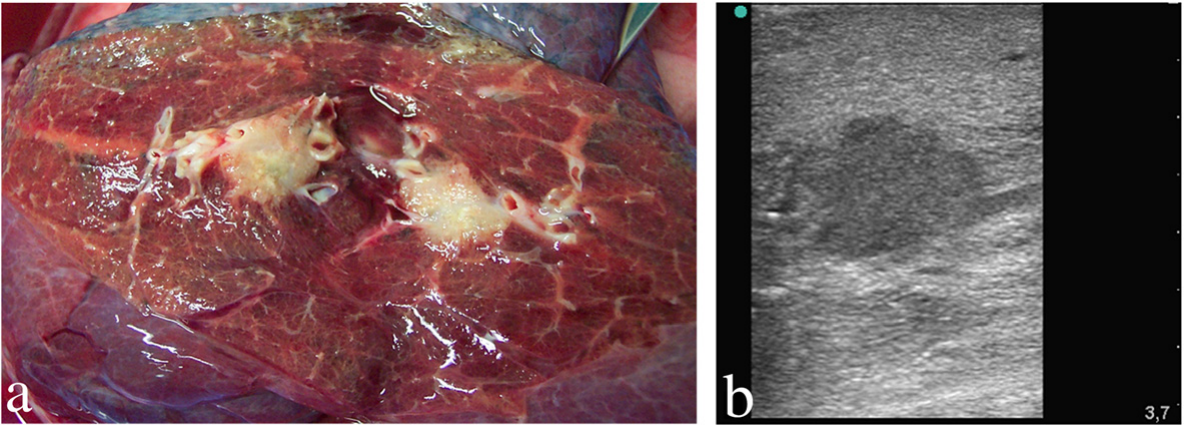
**Sonomorphology of an adenocarcinoma in a flooded human lung lobe. (a)** Macroscopic specimen (cut through the tumour after sonographic examination). **(b)** Sonography of the tumour shows its homogeneous texture, hypoechoicity, and good demarcation from the surrounding normal lung tissue.

**Figure 6:**
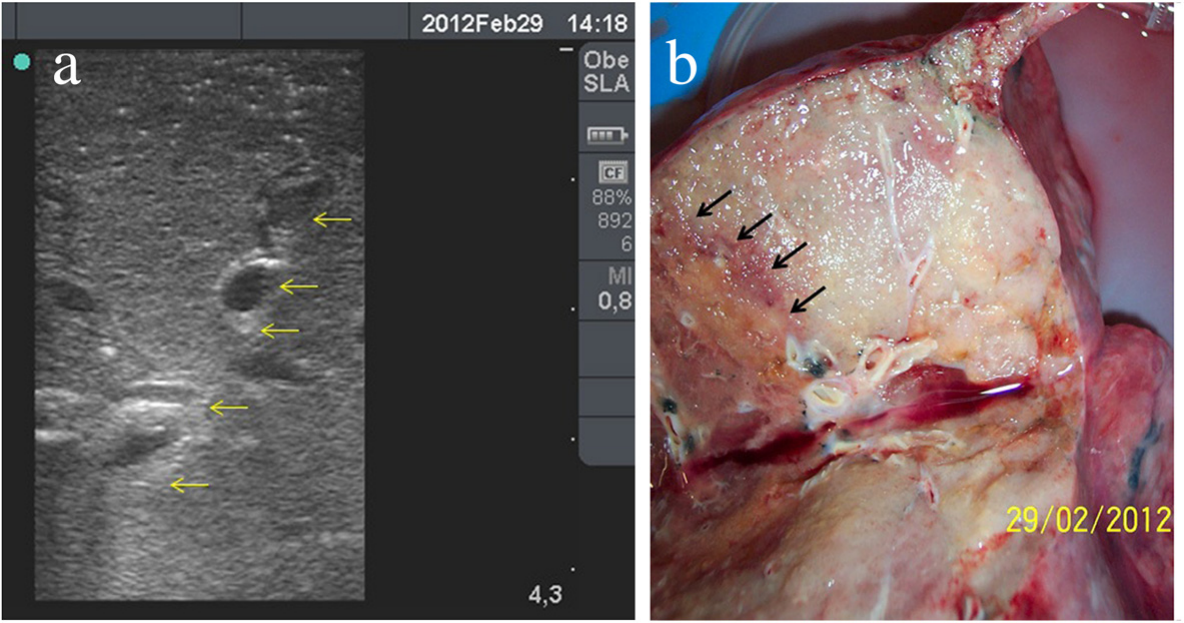
**Sonomorphology of a bronchioloalveolar cell carcinoma in a flooded human lung lobe. (a)** Sonogram of a homogeneous tumour with respect to bronchi and blood vessels. The tumour is slightly hyperechoic compared to surrounding normal lung tissue, making differentiation difficult (arrows mark the border between tumour on the left and normal lung tissue on the right). **(b)** Macroscopic specimen (cut through the tumour after sonographic examination). Tumour border was marked with arrows. The tumour presents as a light grey mass in the right of the photograph.

After flooding, 71.4% (10/14) of the lung lobes could be completely examined by ultrasound, and small amounts of residual gas were observed in 28.6% (4/14) of the cases. Normal lung parenchyma was homogeneous and rich in echogenicity, with a fine-grained structure. Vessels and bronchi differentiated themselves as structures free of echo within the parenchyma, and the bronchial wall displayed its three-layered structure. All tumours were clearly visualised by ultrasound after flooding. Except for bronchioloalveolar carcinoma, all of the other types of non-small cell lung carcinoma were predominantly hypoechoic in comparison to surrounding lung tissue. The tumours were irregularly configured with finger-shaped extensions, and were well-demarcated from the surrounding lung tissue. The typical sonographic images of the main non-small cell lung carcinoma types are as follows: squamous cell carcinoma appears as a predominantly inhomogeneous texture with hypoechoic necrotic areas and a hypoechoic ‘halo’ (Figure 4); adenocarcinoma appears as predominantly homogeneous hypoechoic texture (Figure 5); and large cell carcinoma with neuroendocrine differentiation appears homogeneously hypoechoic with a trabecular or nest-like growth. By contrast, bronchioloalveolar cell carcinoma shows a slightly hyperechoic image. The tumour appears homogenous with respect to the bronchi and vessels, without evidence of infiltration into these structures, and is difficult to differentiate from healthy lung tissue (Figure 6).

**Figure 7:**
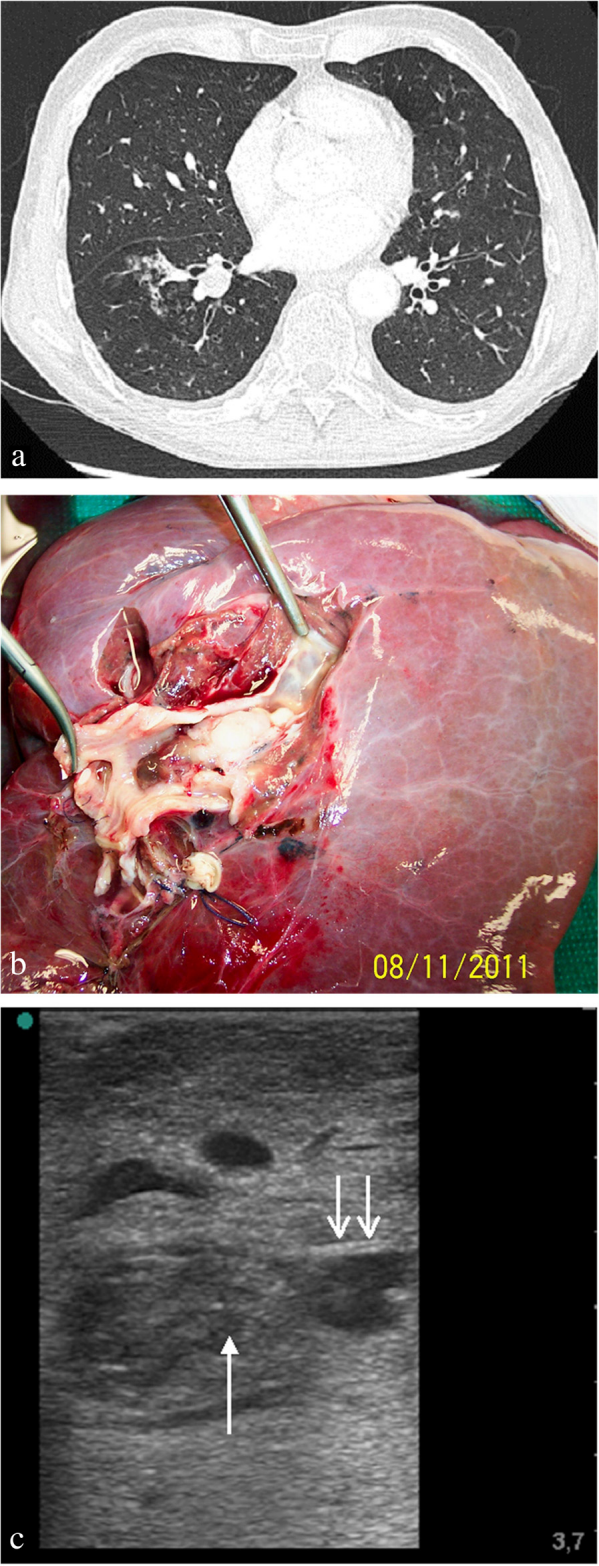
**Sonomorphology of a squamous cell carcinoma with predominantly endobronchial growth in a flooded human lung lobe. (a)** Computed tomography scan of tumour localised to the centre of the right inferior pulmonary lobe**.** There is no evidence of bronchial wall destruction. **(b)** Macroscopic specimen with incised inferior lobar bronchus (after flooding). **(c)** Sonogram shows an inhomogeneous tumour with predominantly endobronchial growth, causing destruction of the bronchial wall (arrow). The normal bronchial wall clearly shows a three-layered structure (two arrows).

When diagnosing bronchus wall infiltration, ultrasound is superior to computed tomography. Transpleural sonography can clearly identify endobronchial tumour growth and destruction of the bronchial wall (Figures 3 and 7).

### *In vivo* ultrasound examination of simulated lung tumours in an animal model

**Figure 8:**
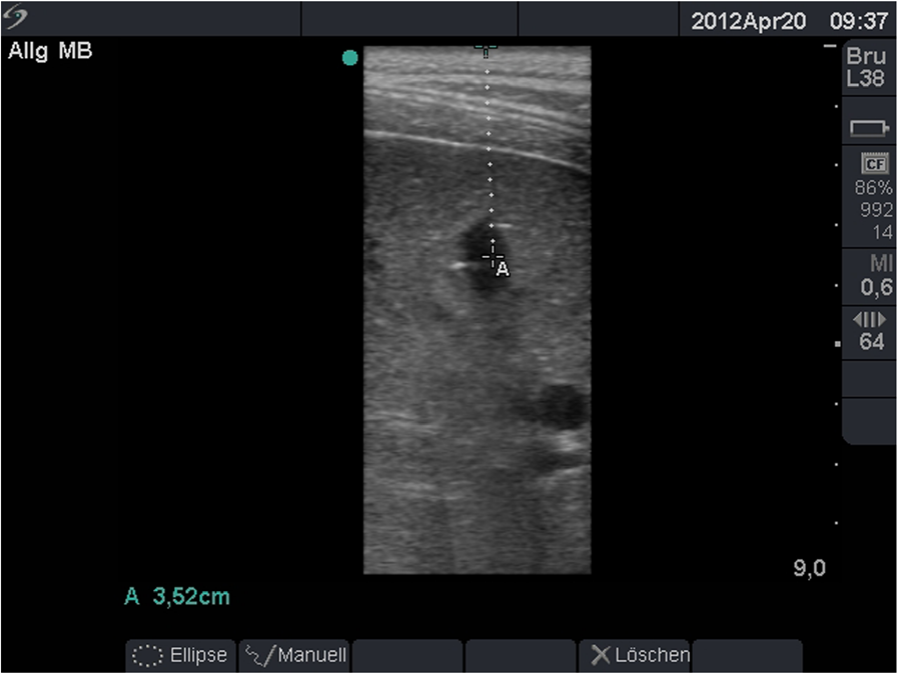
**Ultrasound imaging of the flooded left lung in the *in vivo *porcine model.** Detection of a simulated lung lesion, which appears echo-free with a well-defined margin, at a depth of 3.5 cm. The image shows a pulmonary artery in cross-section without a wall structure, and a bronchus below with a hyperechoic wall at a depth of 6 to 7 cm.

With transthoracic sonography, the flooded animal lung can be completely examined. There is no residual gas to interfere with ultrasound imaging. Pulmonary vessels and bronchi are clearly differentiated. Mediastinal organs such as the heart and thoracic aorta are visible behind the lung. Simulated lung lesions can be detected within the lung lobe. Tumours simulated by Bioglue™ (CryoLife Europa) were completely echo-free with a well-defined margin (Figure 8).

**Figure 9:**
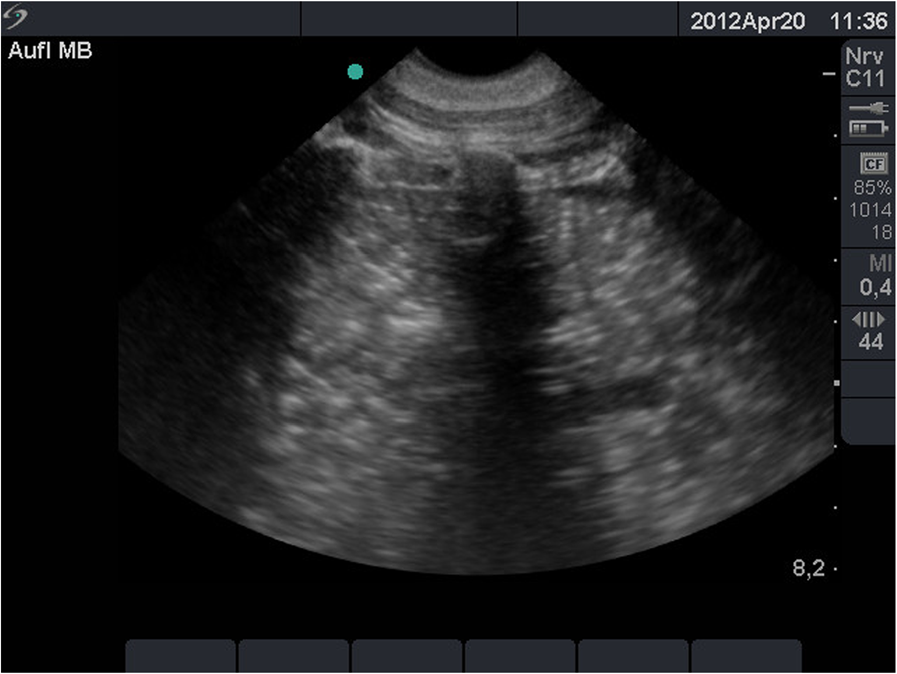
**Transthoracic ultrasound imaging of the flooded left lung immediately after reventilation in the porcine model.** Imaging of the lung is impossible due to many air inclusions. In the centre of the picture, a rib is visible with acoustic shadowing.

Immediately after reventilation of both lungs, sonographic examination shows many air inclusions (Figure 9). After 10 minutes the sound waves were reflected completely by the air-filled lung, and image quality deteriorated considerably.

Both animals survived the procedure without haemodynamic complications. Recovery of the flooding liquid was 35% of the instilled volume in both animals.

## Discussion

Intraoperative sonography is a valuable tool in the surgery of parenchymatous organs. The exact visualisation of the location, size, and spreading of a tumour has a decisive influence on operating strategy [8,9]. In the last five years, sonography has become the imaging technique of choice for guiding intraoperative interventional procedures such as ablation techniques for primary and secondary liver malignancies. Ultrasound gives real-time feedback of the tumour and applicator location, allowing accurate and consistent placement of ablative instruments, as well as evaluation of the developing lesion.

Sonographic imaging of the lung is impossible due to sound reflection due to air content. In comparison with laparoscopic ultrasound-guided tumour detection or interstitial thermotherapy of liver tumours, videothoracoscopic applications of ultrasound are limited. Only in cases of actual tumour infiltration into the pleura, or of bronchial obstruction by the tumour with pneumonic infiltration of the lung tissue up to the pleura, is partial tumour imaging by ultrasound possible [10,11].

We developed a new method for effective ultrasound imaging of the lung. After lung flooding with physiologic saline solution, the lung tissue and lung tumours can be completely visualised, and distinguished, by ultrasound. Tumours are detectable centrally in the lung lobe and differentiate themselves from the surrounding lung parenchyma. Infiltration of the tumour into adjoining functional structures is also identifiable. Furthermore, the surrounding healthy lung parenchyma appears homogeneous and rich in echogenicity, with a fine-grained structure. This is due to multiple scattering at the alveolar septum and water interface. Adenocarcinomas, squamous cell carcinomas, and large cell carcinomas are predominantly hypoechoic in comparison to lung tissue. Bronchioloalveolar cell carcinoma shows a slightly hyperechoic image. The special tumour cell spread inside the alveolar space may result in a higher acoustic impedance.

Tumour detection and complete visualisation by ultrasound is currently inadequate in the atelectatic lung because residual gas in the non-collapsed bronchi strongly hinders complete sonographic imaging. Furthermore, overall organ volume decreases by approximately 80% in the atelectatic lung compared with the ventilated organ. As a result, the true distance between observed tumours and functional lung structures cannot be accurately determined. Our results also show that atelectatic lung tissue and malignant tumour tissue have almost identical echogenicities (that is, isoechoic), and because of this, precise sonographic discrimination between the tumour and healthy lung tissue is not possible.

In animal experiments, a complete one-lung flooding of the non-collapsed lung inside the closed thoracic cavity is possible. The volume of the flooded lung corresponds to the functional residual capacity. Prerequisites for this approach include using a double-lumen tube for safe side-separation, and filling the lung once passively using the gravity of the liquid flowing from an infusion bottle suspended 50 cm above heart level. To minimise the time required for complete sonographic examination, the animal should be placed so that the lung is in the dependent position. Lung flooding enables a complete transthoracic lung sonography. The three-layered bronchial wall structure and blood vessels are clearly differentiated by B-mode sonography. The colour-coded duplex sonography is not helpful because there is no perfusion in the flooded lung. The oscillatory flow occurs in both the vessels and bronchi. Simulated tumours deep inside the parenchyma are easily detected by sonography after lung flooding. Sonography under flooding clearly indicates the spatial relationship between lesions and functional structures.

After passively draining the fluid through the opened tube, complete reventilation of the flooded lung is possible within 30 minutes. The residual saline will be resorbed into the alveoli, assisted by positive-pressure ventilation. All animals survived the procedure. In earlier experiments, we showed that one-lung flooding causes no serious effects on haemodynamic or gas exchange. In comparison with purposeful atelectasis, which is the usual procedure in open and thoracoscopic surgery, lung flooding reduced the pulmonary right-left shunt. This increases the arterial oxygen partial pressure that would otherwise be caused by pulmonary blood flow inhibition [12]. In survival experiments after one-lung flooding, the early postoperative phase after extubation and spontaneous breathing showed a moderate increase in the intrapulmonary shunt fraction that was normalised within 8 hours [13]. A continuous infusion of pentoxifylline increases the partial arterial oxygen pressure and decreases the pulmonary shunt volume during reventilation after flooding, and in the early phase after extubation [14]. Subsequent studies have shown that 1 hour after one-lung flooding, extravascular water in the reventilated lung increases by 5%. After only 24 hours, both the flooded and non-flooded lung no longer differed in their wet-to-dry ratios. The maximum surfactant loss caused by flooding was 47% of the calculated surfactant pool of the respective lung [15]. Finally, histological and immunological investigations demonstrated that one-lung flooding is not associated with destruction of the alveolar texture, atelectasis-provoking surfactant loss, or any irreversible damage to the pulmonary parenchyma [16]. The results available so far in animal studies allow us to conclude that lung flooding over 60 minutes for the purpose of transthoracic or videothoracoscopic lung sonography is safe and justifiable.

The limitations of this new approach are as follows. Lung flooding, both for ultrasound-guided diagnosis and interventional procedures during videothoracoscopy, cannot be performed up to functional residual capacity because this would reduce the space required for satisfactory viewing and endothoracic surgical manipulation. Tumour obstruction of the main lobar bronchi can hinder the fluid filling and sonographic examination of the lung. Severe obstructive lung disease with pulmonary hypertension may be a contraindication for lung flooding.

## Conclusions

Lung flooding enables complete lung sonography and tumour detection, which is otherwise impossible with ultrasound. We created a novel method to use ultrasound for guiding minimally invasive interventional procedures such as thermoablation of lung tumours during videothoracoscopy or open surgery. Lung flooding might be an important and easily accomplished prerequisite for efficiently using high-frequency focused ultrasound to treat lung tumours.

## Abbreviations

CT: Computed tomography; FIO2: Fraction of inspired oxygen; HES: Hydroxyethyl starch; LLL: Left lower lobe; LNM: Lymph node metastasis; MAC: Minimum alveolar concentration; ML: Middle lobe; pCO2: Carbon dioxide partial pressure; RLL: Right lower lobe; RUL: Right upper lobe.

## Competing interests

The authors declare that they have no competing interests, neither financial nor non-financial.

## Authors’ contributions

TGL collected the data and wrote the manuscript. HS and SB performed the anaesthesia. FW was responsible for simulation of lung lesions and ultrasound technique. HS and FW co-wrote the manuscript and discussed the results with TGL. All authors read and approved the manuscript.
